# Iodine status and determinants in adults in Norway – results from a population-based health examination survey (The HUNT Study)

**DOI:** 10.29219/fnr.v68.9761

**Published:** 2024-04-01

**Authors:** Marianne Hope Abel, Torunn Holm Totland, Kristin Holvik, Anne Lise Brantsæter, Steinar Krokstad, Bjørn Olav Åsvold, Haakon E. Meyer

**Affiliations:** 1 Department of Physical Health and Ageing, Norwegian Institute of Public Health, Oslo, Norway; 2 Department of Food Safety, Norwegian Institute of Public Health, Oslo, Norway; 3 HUNT Research Centre, Department of Public Health and Nursing, Faculty of Medicine and Health Sciences, NTNU, Norwegian University of Science and Technology, NTNU, Levanger, Norway; 4 Levanger Hospital, Nord-Trøndelag Hospital Trust, Levanger, Norway; 5 K.G. Jebsen Center for Genetic Epidemiology, Department of Public Health and Nursing, NTNU, Norwegian University of Science and Technology, Trondheim, Norway; 6 Department of Endocrinology, Clinic of Medicine, St. Olavs Hospital, Trondheim University Hospital, Trondheim, Norway; 7 Department of Community Medicine and Global Health, Institute of Health and Society, University of Oslo, Oslo, Norway

**Keywords:** iodine status, urinary iodine concentration, the HUNT-Study, dietary determinants

## Abstract

**Background:**

In Norway, there is a lack of knowledge about the iodine status in the general and older adult population, and there is no established national monitoring programme for iodine. Several studies have indicated that iodine deficiency is prevalent in subgroups of the population. Salt iodisation is currently being considered as a measure to increase the population iodine status. In this cross-sectional study, the aim was to evaluate iodine status and determinants in the adult and older adult population in Mid-Norway, before salt iodisation is likely to be initiated.

**Methods:**

The study sample was a subsample of participants in the fourth wave of the population-based Trøndelag Health Study (HUNT4, 2017–2019) with available spot-urine samples. This subsample included participants with 25–64 years (*n* = 500) and 70–79 years (*n* = 250). The urine samples were analysed for iodine and creatinine. Information on the habitual intake of milk/yoghurt, fish, supplement use, use of thyroid medication and relevant background factors was collected through a general questionnaire. Multivariable quantile regression was used to model differences in the median urinary iodine concentration (UIC) by determinants. Estimates were weighted to match the age and sex distribution of the Norwegian population aged 25–79 years in 2019.

**Results:**

Median UIC was 97 µg/L (95% confidence interval [CI]: 92, 103) indicating borderline iodine deficiency at a group level. The median UIC increased with age, and iodine status was insufficient in participants below age 55 years (median 92 µg/L [95% CI: 85, 99]). Important determinants of UIC were habitual milk/yoghurt intake, daily supplement use and current use of thyroid medication, but not intake of lean or fatty fish. Risk of mild-to-moderate iodine deficiency was seen in those with a low intake of milk/yoghurt, no supplement use and who did not use thyroid medication. No group was identified as being at risk of iodine excess.

**Conclusion:**

Iodine status was adequate in older adults but mildly deficient in adults under 55 years. Milk intake, supplement use and use of thyroid medication are important determinants of iodine intake in Norway.

## Popular scientific summary

- Norway is listed as a country with iodine deficiency by the WHO, but there is a lack of data on iodine status and determinants in different parts of the population.
- This study indicates a low iodine status in adults < 55 years and adequate status in older adults
- Important determinants of iodine status were milk intake, supplement use and use of thyroid medication.
- This study will provide important baseline data before initiation of salt iodisation in Norway.

Iodine is an essential micronutrient, which is required for the synthesis of the thyroid hormones. Deficiency in iodine has a range of potential consequences, commonly referred to as iodine deficiency disorders. Iodine deficiency increases the risk of thyroid disorders and an adaptive swelling of the thyroid gland (goitre) (1). Deficiency has the most severe consequences during foetal development and early childhood, affecting both mental and physical development (1).

Historically, Norway was a country with endemic iodine deficiency, and goitre was common, especially in inland areas where the consumption of saltwater fish was low (2). From the 1950s, iodine was added to the feed for livestock to prevent iodine deficiency in the animals. Iodine passed to the milk, making milk the most important dietary source of iodine in the Norwegian diet. Consequently, iodine deficiency was eradicated in the human population since milk intake was high in the Norwegian diet. During the last decades, trends in the diet characterised by a decrease in milk and fish consumption explain the re-emergence of iodine deficiency (2). Iodine deficiency is well documented in women of childbearing age, pregnant and lactating women, and in vegetarians and vegans (3–7). Deficiency in women of childbearing age is particularly of concern since iodine is important in foetal development (8). In Norway, less is known about the iodine intake of the general and older adult population although some studies indicate that women may be at risk of having a low intake, men have an adequate iodine intake (9, 10) and older adults might be borderline deficient (9, 10).

Currently, Norway is one of few countries left in the world where iodised salt is *not* a significant contributor of iodine in the diet (11). Salt iodisation is the recommended strategy to prevent iodine deficiency according to the World Health Organization (WHO) (12, 13), but uncertainty exists about the risk of iodine excess in groups of the Norwegian population, especially in young children since the range of optimal iodine intake is narrow, and the upper level of safe intake is low (14).

In 2016, The Norwegian Nutrition Council published a report regarding iodine status in Norway declaring an acute need for action to prevent iodine deficiency, and salt iodisation was suggested as the most important measure (15). Following this, the Norwegian Scientific Committee for Food and Environment performed a benefit and risk assessment of salt iodisation and concluded that although salt iodisation would be effective in preventing iodine deficiency in women of childbearing age, it could also impose risk of iodine excess in toddlers (14). Although strongly recommended by the WHO, there is no established monitoring programme for iodine status in Norway. The scenarios for consequences of salt iodisation in Norway were calculated based on dietary surveys, which did not include biological samples but self-reported food intake. Thus, the validity of the estimates is uncertain.

At a population level, the recommended method for assessing iodine status is to measure iodine concentration in spot-urine samples (16). The urinary iodine concentration (UIC) varies a lot by hydration status and by day-to-day variation of iodine intake, and it is therefore of limited value as a measure of iodine status at an individual level. However, at a group level, the median UIC will reflect iodine status. According to the WHO, a median UIC ≥ 100 µg/L in adults is considered to reflect adequate iodine nutrition, whilst median UIC ≥ 300 µg/L indicates a risk of iodine excess. Additionally, the proportion of individuals with UIC < 50 µg/L should be less than 20% (13).

The aim of this study was to evaluate iodine status and important determinants in adults aged 25–79 years from Mid-Norway, and to investigate if subgroups could be defined as being at risk of iodine deficiency or excess.

## Material and methods

### Study sample

The Trøndelag Health Study (the HUNT Study) is a population-based cohort study of the adult population in Trøndelag County, Norway (17). The study has been running in Nord-Trøndelag since 1984 and is designed to cover a broad range of health-related topics through repeated surveys with questionnaires, interviews, clinical examinations, laboratory measurements and storage of biological samples. All residents ≥ 20 years of age in the Nord-Trøndelag were invited to the fourth wave of the HUNT study (HUNT4) in 2017–2019. The participation rate of the HUNT4 in Nord-Trøndelag was 49% in men and 59% in women, and the total number of participants was 56,042 (17).

Spot-urine samples were collected from a subsample of participants in the HUNT4 survey (*n* = 26,961). The subsample consisted of participants who had previously participated in the HUNT2 and 3 Microalbuminuria Study (~60% of participants), and a random sample of other HUNT4 participants from large and some small municipalities, limited due to logistic factors. Participants with known kidney disease, heart failure or cerebral stroke were excluded.

The study sample for our study (*n* = 750) was randomly selected amongst eligible HUNT4-participants who had donated urine samples, stratified by age and sex (125 woman and 125 men for each of the age-groups 25–44, 45–64 and 70–79 years). This study sample was purposely drawn to match the age and sex distribution in a previous study sample from HUNT3 (2006–2008), where the main aim was to measure changes in urinary sodium concentration.

### Analyses of urinary creatinine and iodine concentration

Participants who showed up at the clinical examination were asked if they could donate a urine sample. The samples were non-fasting and from any time during the day (09.00 – 20.00 h). After sampling, the urine samples were immediately cooled down to 4°C, transported to HUNT Biobank the same day and frozen to −80°C the following day for storage. The analyses of creatinine were done at Levanger Hospital with Architect ci8200, using an enzymatic assay. The measurement range was 220 – 35,360 µmol/L, and the total assay coefficient of variation (CV) was 24% (5,790 µmol/L).

Analysis of urine iodine concentration was performed at the Hormone Laboratory, Oslo University Hospital, Norway. Iodine concentration was measured colorimetrically by the Sandell–Kolthoff’s reaction based on the catalytic effect of iodine on the redox reaction between arsenic and cerium after ammonium persulfate digestion of the samples. The total CVs were 15% at iodine concentration of 57 µg/L, 10% at 97 µg/L and 5.4% at 291 µg/L. The Hormone Laboratory is accredited as a testing laboratory by Norwegian Accreditation according to the standard NS-EN ISO/IEC 17025, with a Registration number TEST 099.

#### Estimated 24-h urinary excretion (24 h UIE) and daily iodine intake

Daily urinary iodine excretion (24 h UIE) was estimated based on a method proposed by Johner et al. (18). The method includes a prediction model for estimating 24 h urinary creatinine concentration based on information on sex, age, BMI and bodyweight. This model was developed using linear regression in a representative sample of the German population with available 24 h urinary creatinine measurements (*n* = 1,463, age: 20–79 years).

Prediction equation for 24 h creatinine excretion (mmol per day) = e^(1.9539 + 0.1681 × sex − 0.0027 ×age (years) + 0.0129 × weight (kg) − 0.0129 × BMI (kg/m2))^.

Twenty-four-hour UIE was estimated using the following equation: 24 h UIE (µg/24 h) = (UIC (µg/L)/Urinary creatinine (mmol/L)) × (estimated 24 h creatinine excretion (mmol/day)).

To convert the estimated 24 h UIE to estimated iodine intake, the UIE was divided by 0.92. This factor reflects the bioavailability of iodine since ~92% of ingested iodine is excreted in the urine (16).

Data on all covariates needed to estimate 24 h UIE, and iodine intake was available for *n* = 745 of 750 participants.

### Determinants and background factors

Information on participant’s age, sex and marital status was obtained from the Population Register of Norway. All participants in HUNT4 filled in a general questionnaire containing 48 questions on health, medication, lifestyle, well-being, childhood and some background factors (e.g. education and income). In our study, we used the self-reported data from this questionnaire on habitual intake of milk (glasses per week or day), yoghurt (portions per week or day), lean fish (fish fillet, for example cod or saithe, frequency per week), fatty fish (e.g. salmon, trout, herring or mackerel, as dinner or spread, frequency per week), supplement use (calcium, omega-3/cod liver oil or other vitamin and/or mineral supplements and current frequency of use for each), current use of thyroid medication (yes/no), current smoking (no, sometimes and daily), completed education (primary school, secondary school, college/university < 4 years and college/university ≥ 4 years) and household income (in 1,000 NOK: ≤ 450, 451–750, 751–1,000 and > 1,000). The self-reported supplement use may have included supplements with or without iodine, and the dosage was not specified. Body weight and height were measured at the clinical examination.

### Statistical analyses

Statistical analyses were performed in STATA (version 16.0; Stata Corp., College Station, TX).

Population weights were applied in *all* the analyses to standardise the results to the age and sex distribution of the Norwegian population aged 25–79 years in 2019. Population weights were applied by sex and age group (25–29, 30–39, 40–49, 50–59, 60–69 and 70–79 years).

Associations between independent variables and median UIC or median UIE were estimated by quantile regression adjusting for relevant covariates (using the command qreg in STATA). Modelling the median by quantile regression was chosen as a method both since the outcome measures were highly skewed and because the median value for urinary iodine is used to define cut-off-values for iodine deficiency and excess by the WHO. Modelling the median also provides estimates that are easier to interpret than, for example, modelling the log of the outcome by linear regression. Differences in median UIC may be caused by differences in iodine intake, in iodine absorption or in 24 h urine volume, for example, by age, sex or BMI. To control for differences in 24 h urine volume, we additionally used median estimated UIE as an outcome. We modelled the crude associations, and associations adjusted for relevant background factors and for dietary determinants. Participants with extreme values of urinary iodine were excluded from the regression models (i.e. UIC > 1,000 µg/L [*n* = 2], estimated UIE > 1,000 µg/24 h [*n* = 4] or estimated iodine intake > 1,200 µg/day [*n* = 2]).

Only complete cases were included in the respective models. There were no missing values for the urinary measures, and few for background factors (0–1.1%, see Table 1), but there were some missing values for the intake of milk/yoghurt (4.8%) and lean fish (0.4%). Participants who had not reported use of thyroid medication or vitamin/mineral supplements were coded as non-users.

**Table 1:** Participant characteristics and median spot-urinary iodine concentration (UIC). The HUNT Study (HUNT4 2017–19), Norway.

Characteristics	Study population	UIC^a^ µg/L
		median (95% CI)
**Study sample, *n* (%)**	750 (100)	97 (93, 103)
Men 25–49 years	153 (20)	91 (81–101)
Men 50–64 years	97 (13)	110 (92–128)
Men 70–79 years	125 (17)	118 (104–131)
Women 25–49 years	146 (19)	95 (82–108)
Women 50–64 years	104 (14)	90 (80–100)
Women 70–79 years	125 (17)	118 (102–134)
**BMI, mean (SD), kg/m** ^2^	27.2 (4.7)	
< 18.5	7 (0.9)	48 (32, 64)
18.5–24.9	256 (34)	91 (82, 100)
25–29.9	312 (42)	97 (88, 107)
30–34.9	130 (17)	109 (96, 122)
35+	42 (5.6)	105 (60, 150)
Missing	3 (0.4)	
**Education, completed**		
Primary school	190 (25)	103 (89, 116)
Secondary school	230 (31)	92 (83, 102)
College/University < 4 years	182 (24)	100 (91, 109)
College/University ≥ 4 years	146 (19)	99 (80, 118)
Missing	2 (0.3)	
**Marital status**		
Not married	206 (27)	94 (82, 106)
Married	430 (57)	95 (88, 102)
Widow	40 (5.3)	110 (94, 126)
Divorced or separated	73 (9.7)	110 (89, 131)
Missing	1 (0.1)	
**Current smoking**		
No	670 (89)	99 (92, 105)
Occasionally	24 (3.2)	89 (62, 116)
Daily	54 (7.2)	84 (62, 105)
Missing	2 (0.3)	
**Household income (1,000 NOK)**		
≤ 450	216 (29)	105 (95, 115)
451–750	242 (32)	99 (87, 110)
751–1,000	151 (20)	95 (83, 107)
> 1,000	133 (18)	91 (77, 105)
Missing	8 (1.1)	

a The median UIC was weighted to match the sex and age distribution of the Norwegian population aged 25–79 years in 2019.

Confidence intervals (CIs) for median values were calculated using a binomial method that makes no assumptions about the underlying distribution of the variable (command centile in STATA). A *P*-value < 0.05 was considered statistically significant.

### Ethics

The study was approved by the Regional Committee for Medical Research Ethics Mid-Norway (REK midt 20339).

## Results

Background characteristics of the study sample (*n* = 750) and UICs in subgroups are shown in Table 1.

**Fig. 1:**
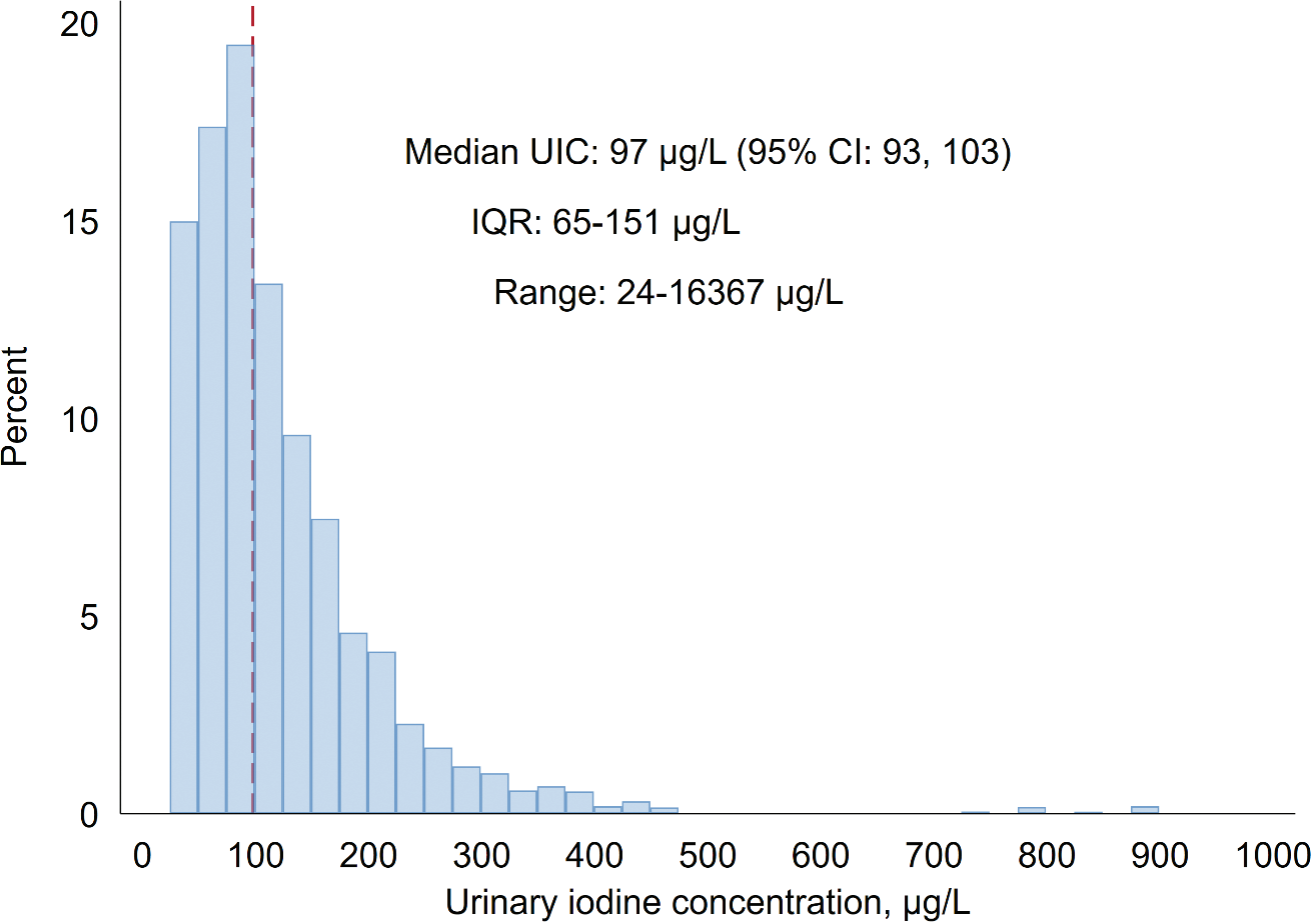
Population-weighted distribution of spot-urinary iodine concentration (UIC, *n* = 750). Values higher than 1,000 µg/L (*n* = 2) are omitted from the figure. The red line indicates the median value. The HUNT Study (HUNT4 2017–19), Norway.

The distribution of UIC is shown in Fig. 1. The population-weighted median UIC was 97 µg/L (95% CI: 93, 103), indicating borderline iodine deficiency at the group level. The spot-UIC was < 50 µg/L in 16% (95% CI: 13, 19). Median estimated iodine intake, calculated from UIC, urinary creatinine, age, sex, BMI and body weight, was 171 µg/day (95% CI: 160, 183). It was 165 µg/day in women (95% CI: 149, 182) and 179 µg/day in men (95% CI: 162, 196).

### Determinants of urinary iodine

Median UIC increased with age (*P* = 0.002) but did not differ substantially by sex or by income or marital status (Table 2). Education was associated with UIC but not estimated 24 h UIE, and BMI was positively associated with UIC and estimated 24 h UIE after controlling for background factors and dietary determinants (Table 2). Participants who reported current smoking had lower UIC and lower estimated 24 h UIE, also after controlling for background factors and dietary determinants (Table 2).

**Table 2:** Estimated difference in median urinary iodine by background factors*.

Subgroup	Study population *n* (%)	Median spot-UIC, µg/L			Median estimated UIE, µg/24 h		
		Model 0^a^	Model 1^b^	Model 2^c^	Model 0^a^	Model 1^b^	Model 2^c^
		Beta (95 % CI)	Beta (95 % CI)	Beta (95 % CI)	Beta (95 % CI)	Beta (95 % CI)	Beta (95 % CI)
Men	375 (50)	Ref.	Ref.	Ref.	Ref.	Ref.	Ref.
Women	375 (50)	−4 (−16, 8)	−3 (−13, 6)	−3 (−12, 6)	−14 (−35, 8)	−11 (−28, 6)	−15 (−28, −2)
		*P* = 0.53	*P* = 0.48	*P* = 0.58	*P* = 0.21	*P* = 0.21	*P* = 0.027
**Age, years**							
25–34	119 (16)	Ref.	Ref.	Ref.	Ref.	Ref.	Ref.
35–44	131 (17)	−4 (−21, 14)	1 (−19, 21)	3 (−15, 21)	−12 (−42, 17)	−20 (−47, 8)	−6 (−31, 20)
45–54	99 (13)	−5 (−25, 15)	−5 (−20, 9)	−10 (−25, 4)	19 (−9, 48)	5 (−24, 33)	−3 (−29, 23)
55–64	151 (20)	14 (−2, 30)	20 (6, 34)	11 (−3, 25)	31 (0, 63)	18 (−8, 45)	8 (−18, 35)
70–79	250 (33)	24 (10, 39)	27 (12, 41)	20 (7, 32)	40 (14, 66)	24 (−2, 49)	19 (−10, 48)
		*P* = 0.002	*P* < 0.001	*P* < 0.001	*P* < 0.001	*P* = 0.015	*P* = 0.16
**BMI, kg/m** ^2^							
< 25	263 (35)	Ref.	Ref.	Ref.	Ref.	Ref.	Ref.
25–29.9	312 (42)	6 (−6, 19)	3 (−8, 13)	5 (−7, 17)	5 (−19, 29)	9 (−9, 27)	18 (4, 31)
≥ 30	172 (23)	16 (1, 31)	14 (2, 27)	14 (2, 26)	18 (−15, 51)	25 (0, 50)	37 (18, 55)
		*P* = 0.10	*P* = 0.065	*P* = 0.039	*P* = 0.67	*P* = 0.15	*P* < 0.001
**Education, completed**							
Primary school	190 (25)	Ref.	Ref.	Ref.	Ref.	Ref.	Ref.
Secondary school	230 (31)	−11 (−28, 6)	−1 (−14, 13)	6 (−6, 18)	−17 (−48, 14)	−9 (−29, 12)	7 (−12, 26)
College/University < 4 years	182 (24)	−3(−19, 14)	11 (−1, 24)	15 (1, 29)	8 (−28, 43)	18 (−8, 44)	19 (1, 37)
College/University ≥ 4 years	146 (19)	−4 (−27, 20)	20 (2, 38)	14 (−2, 30)	−2 (−33, 29)	17 (−12, 45)	29 (5, 53)
		*P* = 0.49	*P* = 0.048	*P* = 0.14	*P* = 0.37	*P* = 0.10	*P* = 0.051
**Marital status**							
Married	430 (57)	Ref.	Ref.	Ref.	Ref.	Ref.	Ref.
Not married/divorced/widowed	319 (43)	8 (−4, 20)	12 (0, 24)	11 (0, 21)	−27 (−49, −5)	−6 (−24, 12)	−10 (−24, 4)
		*P* = 0.21	*P* = 0.041	*P =* 0.050	*P* = 0.015	*P* = 0.52	*P* = 0.16
**Current smoking**							
No	670 (89)	Ref.	Ref.	Ref.	Ref.	Ref.	Ref.
Occasionally	24 (3.2)	−10 (−38, 18)	−2 (−13, 9)	11 (−7, 29)	−55 (−126, 16)	0 (−99, 98)	10 (−40, 61)
Daily	54 (7.2)	−15 (−38, 7)	−17 (−30, −4)	−18 (−31, −5)	−66 (−85, −47)	−64 (−83, −45)	−55 (−76, −34)
		*P* = 0.34	*P* = 0.036	*P* = 0.008	*P* < 0.001	*P* < 0.001	*P* < 0.001
**Household income (1,000 NOK)**							
≤ 450	216 (29)	Ref.	Ref.	Ref.	Ref.	Ref.	Ref.
451–750	242 (32)	−6 (−21, 9)	−3 (−15, 8)	2 (−11, 14)	39 (7, 71)	26 (4, 48)	18 (−3, 40)
751–1,000	151 (20)	−11 (−26, 3)	−6 (−24, 12)	−9 (−24, 5)	25 (−3, 53)	16 (−8, 39)	16 (−7, 39)
> 1,000	133 (18)	−14 (−31, 3)	−11 (−27, 4)	−10 (−27, 6)	36 (3, 68)	11 (−20, 43)	31 (6, 56)
		*P* = 0.31	*P* = 0.53	*P* = 0.28	*P* = 0.069	*P* = 0.13	*P* = 0.068

* Differences in median urinary iodine are estimated based on quantile regression, and estimates are population weighted. Complete case analyses. Urinary iodine-values exceeding 1,000 µg/L (*n* = 2) or 1,000 µg/24 h (*n* = 4) were omitted.

a Model 0: Crude – not adjusted for covariates (*n* = 733–748).

b Model 1: Mutually adjusted for all covariates in Table 1 (*n* = 732 for UIC/*n* = 728 for UIE).

c Model 2: Similar as Model 1, and additionally adjusted for thyroid medication, supplement use, milk/yoghurt intake, lean fish intake and fatty fish intake (*n* = 695 for UIC/*n* = 691 for UIE).

**Fig. 2:**
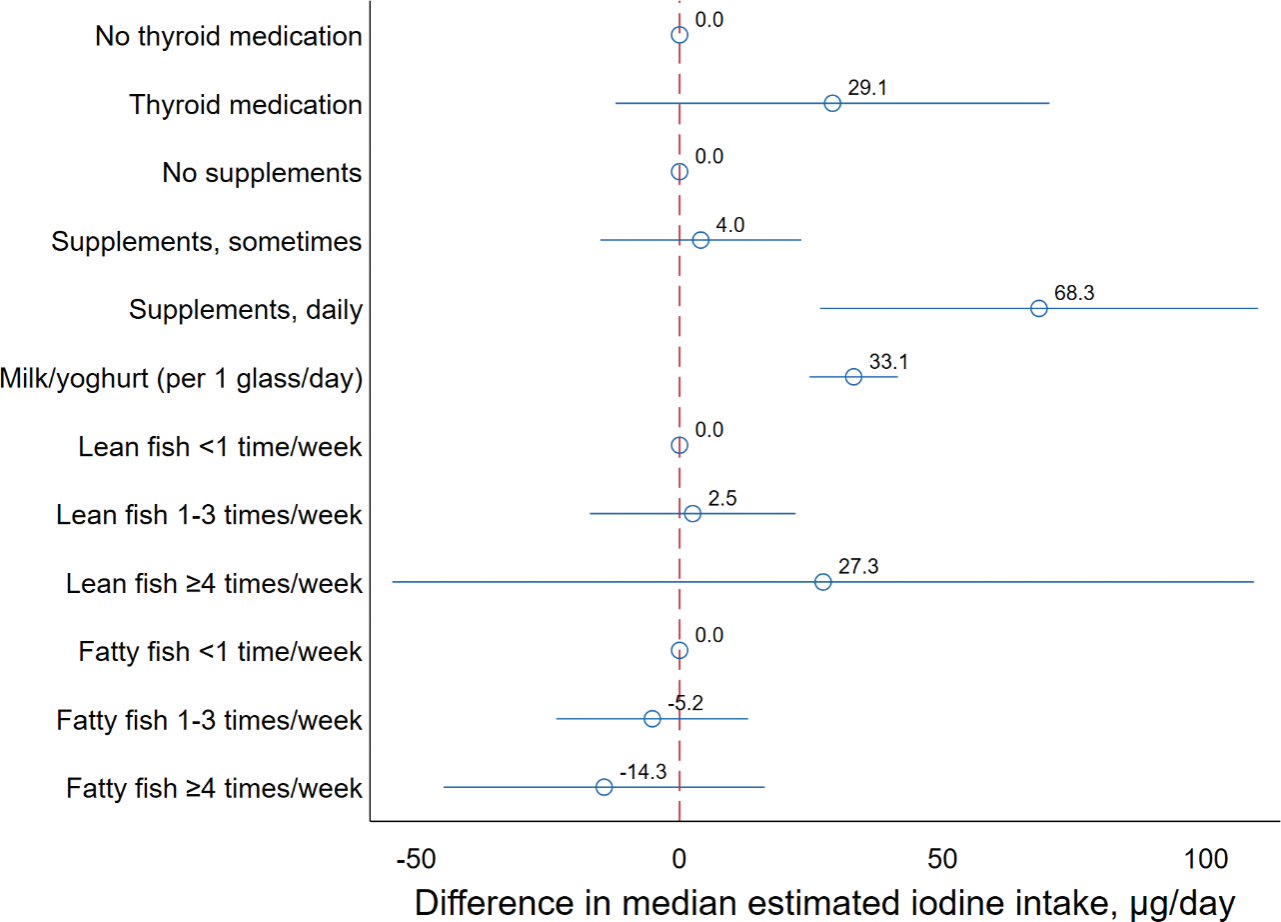
Plot showing the change in median estimated iodine intake by predictors. Estimated betas and 95% confidence intervals are from quantile regression mutually adjusting for all factors, and in addition adjusting for age, sex, BMI and population weighted. The reference level (beta = 0) was set to: no use of thyroid medication, no supplement use, lean fish < 1 time/week and fatty fish < 1 time/week. Iodine intake was estimated based on spot-urinary iodine and creatinine, age, sex, BMI and body weight. Participants with estimated iodine intake > 1,200 µg/day (*n* = 2) were excluded from the model.

Intake of milk/yoghurt, use of thyroid medication and daily use of a non-specified vitamin and/or mineral supplements (other than cod liver oil or other omega-3 supplements and calcium) were associated with having a higher UIC and higher estimated 24 h UIE (Table 3). Intake of lean and fatty fish was not significantly associated with UIC (Table 3). Modelling milk/yoghurt as a continuous variable showed that for each glass of milk/yoghurt per day, the estimated increase in median UIC was 10 µg/L (95% CI: 5, 15, *P* < 0.001), adjusting for age, sex, BMI, supplement use, thyroid medication and intake of lean and fatty fish. The corresponding increase in estimated median iodine intake per daily glass of milk/yoghurt was 33 µg/day (95% CI: 25, 41, *P* < 0.001) (Fig. 2). The median intake of milk/yoghurt was 1 glass/day (interquartile range (IQR): 0.5, 2).

**Table 3:** Estimated difference in median urinary iodine* by relevant dietary factors, supplement use and thyroid medication, – the major determinants of iodine intake in Norway.

Subgroup	Study population *n* (%)	UIC, µg/L			Estimated UIE, µg/24 h		
		Model 0^a^	Model 1^b^	Model 2^c^	Model 0^a^	Model 1^b^	Model 2^c^
		Beta (95 % CI)	Beta (95 % CI)	Beta (95 % CI)	Beta (95 % CI)	Beta (95 % CI)	Beta (95 % CI)
Thyroid medication – current use							
Not reported	704 (94)	Ref.	Ref.	Ref.	Ref.	Ref.	Ref.
Yes	46 (6.1)	56 (33, 79)	46 (16, 76)	33 (19, 47)	50 (0, 100)	41 (−12, 93)	33 (−3, 69)
		*P* < 0.001	*P* = 0.003	*P* < 0.001	*P* = 0.050	*P* = 0.13	*P* = 0.070
Vitamin/mineral supplement^d^							
Never	363 (48)	Ref.	Ref.	Ref.	Ref.	Ref.	Ref.
Occasionally or in periods	227 (30)	0 (−13, 13)	3 (−11, 16)	3 (−6, 13)	8 (−14, 30)	12 (−10, 35)	3 (−13, 18)
Daily	160 (21)	27 (5, 49)	27 (4, 50)	25 (4, 46)	70 (35, 104)	65 (29, 101)	63 (28, 98)
		*P* = 0.053	*P* = 0.071	*P* = 0.073	*P* < 0.001	*P* = 0.002	*P* = 0.002
Milk/yoghurt^e^							
Seldom/never	74 (9.9)	Ref.	Ref.	Ref.	Ref.	Ref.	Ref.
1–6 glasses/week	132 (18)	9 (−11, 29)	15 (−8, 37)	2 (−12, 16)	4 (−19, 27)	0 (−24, 24)	8 (−8, 23)
1 glass/day	285 (38)	23 (3, 43)	25 (4, 45)	22 (7, 36)	33 (12, 54)	32 (10, 54)	42 (24, 59)
2–3 glasses/day	186 (25)	41 (19, 62)	44 (21, 66)	37 (21, 53)	82 (58, 105)	77 (56, 98)	81 (63, 98)
≥ 4 glasses/day	37 (4.9)	35 (−4, 74)	39 (−3, 81)	40 (4, 76)	137 (87, 187)	126 (71, 182)	134 (84, 185)
		*P* < 0.001	*P* = 0.001	*P* < 0.001	*P* < 0.001	*P* < 0.001	*P* < 0.001
Lean fish filet^*^							
< 1 time/week	262 (35)	Ref.	Ref.	Ref.	Ref.	Ref.	Ref.
1–3 times/week	445 (59)	11 (−1, 23)	5 (−8, 18)	7 (−4, 19)	25 (1, 48)	17 (−7, 40)	2 (−14, 19)
≥ 4 times/week	40 (5.3)	13 (−15, 40)	4 (−17, 25)	14 (−13, 41)	27 (−69, 124)	9 (−88, 107)	61 (4, 118)
		*P* = 0.16	*P* = 0.71	*P* = 0.39	*P* = 0.11	*P* = 0.39	*P* = 0.11
Fatty fish (dinner and spread)							
< 1 time/week	227 (30)	Ref.	Ref.	Ref.	Ref.	Ref.	Ref.
1–3 times/week	472 (63)	−1 (−13, 11)	−5 (−17, 7)	−18 (−29, −7)	20 (−2, 42)	7 (−16, 29)	−4 (−19, 10)
≥ 4 times/week	48 (6.4)	6 (−27, 40)	−8 (−42, 26)	−23 (−45, −2)	40 (−6, 86)	23 (−25, 71)	−13 (−32, 7)
		*P* = 0.89	*P* = 0.70	*P* = 0.004	*P* = 0.10	*P* = 0.61	*P* = 0.43

* Changes in median urinary iodine are estimated based on quantile regression and estimates are population weighted. Complete case analyses. Participants with UIC > 1,000 µg/L (*n* = 2) and UIE > 1,000 µg/24 h (*n* = 4) were excluded from the models.

a Model 0: Crude – not adjusted for covariates (*n* = 706–748).

b Model 1: Adjusted for age (restricted cubic splines with 3 knots), sex and BMI (restricted cubic splines with 3 knots) (*n* = 706–745).

c Model 2: Similar as Model 1 and additionally mutually adjusted for all covariates listed in the table (*n* = 706 for UIC and *n* = 702 for UIE).

d Any other vitamin and/or mineral supplement other than cod liver oil, omega 3-supplement or calcium supplement.

e Non-fermented and fermented milk for drinking plus yoghurt (3 glasses = 0.5 L).

The age group 70–79 years had a higher median UIC than younger participants (median 118 µg/L vs. 94 µg/L in the age group 25–64 years, *P* < 0.001), and a higher estimated UIE. Comparing the two groups, the older adults were more likely to use thyroid medication (11% vs. 4%, *P* = 0.001), to report a daily vitamin/mineral supplement (29% vs. 18%, *P* < 0.001), to eat lean fish at least once a week (80% vs. 58%, *P* < 0.001) and to eat fatty fish at least once a week (76% vs. 66%, *P* = 0.005). There was no difference in milk/yoghurt intake, and the mean intake in both groups was 1.5 glasses/day. The differences in 24 h UIE by age groups were attenuated and no longer statistically significant after controlling for these iodine sources (Table 2).

### Risk of iodine insufficiency or excess

Participants aged 25–54 years (*n* = 349) had a median UIC < 100 µg/L (median: 93 µg/L [95% CI: 85, 99]), indicating an insufficient intake, and 21% had < 50 µg/L (95% CI: 17, 25). The median estimated iodine intake in this age group was 156 µg/day (95% CI: 147, 173).

Participants of all ages who consumed less than 1 glass of milk/yoghurt per day and did not use a daily supplement or thyroid medication (*n* = 152) had a median UIC of 75 µg/L (95% CI: 66, 84), and their median estimated iodine intake was 123 µg/day (95% CI: 109, 137).

Based on the available information, no consumption pattern could be identified to be associated with risk of iodine excess (i.e. median UIC ≥ 300 µg/L or iodine intake ≥ 600 µg/day). Model 2 described in Table 3 was used to estimate median UIC in adults who use thyroid medication, a daily supplement, consume ≥ 4 glasses of milk/yoghurt per day and eat lean fish ≥ 4 times a week. Estimated median UIC in this hypothetical high-intake group was 177 µg/L (95% CI: 129, 225), and estimated median iodine intake was 411 µg/day (95% CI: 320, 502).

## Discussion

The main findings in this study were that iodine status in older adults was adequate at a group level, whereas adults under age 55 years had mild iodine deficiency according to the current WHO criteria. Intake of milk/yoghurt, daily supplement use and use of thyroid medication were important determinants of iodine status and could largely explain the age differences.

Although this current study was not performed on a country-representative sample, the results are likely to provide reasonably good estimates of iodine status in adults and older adults in Norway. Results from the National Public Health Survey, 2020, show that the self-reported intake of milk, fish and supplements containing iodine was not different in Trøndelag, the region where this study was conducted, compared to the country averages (19). The iodine status measured in our study is close to what has been measured in two previous studies of the general adult population in Norway. In the Tromsø study, a population-based health study in Northern Norway (year 2015–2016, *n* = 463, age: 40–69 years), the median UIC was 88 µg/L (10). In comparison, it was 97 µg/L in our study, and 95 µg/L (95% CI: 88, 102) if the study sample was restricted to the comparable age range (40–69 years). In Norkost 3, a National dietary survey (year 2010–2011, *n* = 1,787, age: 18–70 years), iodine intake was calculated based on two 24 h dietary recall interviews (9). The median iodine intake was 151 µg/day, and in our study, the estimated median iodine intake was 171 µg/day or 170 µg/day (95% CI: 157, 182) if excluding participants > 70 years. Our study contributes with new knowledge regarding iodine status in the age group 70–79 years.

Our analyses show that milk intake and supplement use are important determinants of iodine status. For every daily glass of milk, the estimated iodine intake increased by 33 µg. This corresponds well to the iodine concentration in Norwegian milk, which currently is in the range 15–20 µg/dL (20). The median milk/yoghurt intake was one glass/day, and this is below the recommendation in Norway of three portions of dairy products a day where at least two portions should be milk/yoghurt (21). Reporting daily supplement use, other than omega 3-supplements and calcium supplements, was associated with a 68 µg/day increase in median estimated iodine intake. The iodine-containing supplements on the Norwegian market typically contain 75–225 µg per daily dose, but not *all* supplements contain iodine. Our result reflects the increase in iodine intake in the average user of the different supplements, and, thus, the iodine intake of an iodine supplement user is expected to be higher depending on dose.

Fish intake was not significantly associated with urinary iodine in our study, but a frequent intake of lean fish (≥ 4 times a week) was associated with a non-significant increase in estimated iodine intake of 27 µg/day, compared to eating lean fish less than once a week. Lean fish has a natural high content of iodine and can be an important food source if eaten regularly (9, 22). It may be difficult, however, to measure the effect of lean fish on iodine status by a single spot-urine sample since the urine sample only provides a snapshot of the recent iodine intake within the last few hours. A single dinner with lean fish (200 g) can provide typically about 50–800 µg of iodine (20), which is a significant contribution compared to the daily recommended intake of 150 µg iodine/day. However, the consumption of fish in Norway is lower than recommended, and younger adults have a lower consumption on average than older adults (19). Fatty fish has a low iodine content (e.g. 5 µg/100 g in farmed salmon) and was not associated with urinary iodine in our study, as expected.

We found no clear association between socioeconomic factors and urinary iodine, and this finding is in line with what has previously been described in a study including nearly 80,000 pregnant mothers in The Norwegian Mother, Father and Child Cohort Study, where iodine intake was calculated from a food frequency questionnaire (23). Thus, socioeconomic status is most likely not an important determinant of iodine intake in Norway.

Furthermore, we found no difference between men and women, in either UIC or 24 h UIE. This is in contrast with findings from the National dietary survey, Norkost 3, where men had higher calculated iodine intake than women (median 176 µg/day vs. 130 µg/day) (9). Also, in the Tromsø Study, where iodine was measured in 24 h urine samples and by an FFQ, the iodine excretion was higher in men compared to women (median UIE was 145 µg/24 h vs. 111 µg/24 h) (10). Results from the FFQ showed that the difference could be explained by a higher overall food intake in men compared to women. In our study, we had only one single spot-urine sample for estimating habitual iodine intake, and the measurement error is therefore quite large. Thus, we might have needed a larger sample size to document a potential difference between men and women in our study.

BMI was positively associated with 24 h UIE when controlling for background factors and dietary determinants (Table 2). This finding probably reflects that the total food intake increases with BMI and, consequently, so does the intake of iodine from other food sources like eggs, cheese, brown cheese and more.

Smoking, on the other hand, was associated with a lower UIC and a lower estimated 24 h UIE when controlling for other determinants (Table 2). Daily smoking was associated with an estimated reduction in median UIE of 55 µg/24 h, which is quite a substantial reduction since the median UIE for the whole study population was 158 µg/24 h. This phenomenon might be partly caused by the inhibition of the iodine transporter by thiocyanate, a degradation product of cyanide from tobacco smoke (24, 25). Inhibition of the iodine transporter probably decreases iodine uptake from the gastrointestinal tract and, thus, decreases the bioavailability of ingested iodine. Several studies have found that goitre is more prevalent in smokers compared to non-smokers in iodine deficient populations (24, 26). Importantly, smoking tobacco is also associated with increased creatinine excretion in the urine (27), which may produce an artificially low 24-h UIE estimation in smokers in our study. Thus, to evaluate the effect of smoking on iodine uptake, one should collect 24-h urine samples in addition to data on other iodine status determinants.

### Risk of iodine deficiency

The results from our study suggest that people with a low iodine status can easily be identified if you have data on their milk intake, supplement use, smoking status and use of thyroid medication. In Norway, large population groups fall below the WHO cut-off defining iodine deficiency, including adults under 55 years in our study. However, this may mostly be of concern for women of childbearing age since pregnancy and lactation requires extra iodine, and studies indicate that iodine intake ideally should be higher (i.e. median UIC > 100 µg/L) for some time before conception (28). For other adults, the WHO cut-off defining deficiency might be set too high, and it is discussed whether it should be lowered to median UIC < 60–70 µg/L instead of < 100 µg/L (16). In our study, no group could be identified that fell below this level. Other studies in Norway have shown that there are certain groups at risk of iodine deficiency, particularly vegetarians and vegans (7), women of childbearing age (6) and pregnant and lactating women (5, 29, 30).

### Potential risk of iodine excess

In this study, we could not identify any consumption pattern that would put people at risk of having an excess iodine intake (i.e. median UIC ≥ 300 µg/L or iodine intake ≥ 600 µg/day). Even a very high intake of milk/yoghurt (≥ 4 glasses/day) and lean fish (≥ 4 times per week), combined with daily supplement use and use of thyroid medication gave an estimated median UIC and estimated iodine intake well below the excess range. However, we did not have detailed data on iodine supplement use and used information on ‘vitamin/mineral-supplements other than omega-3 supplements and calcium supplements’ as a proxy. Daily use of an iodine-containing supplement or seaweed products could therefore result in a higher UIC than what we have estimated, depending on iodine content.

Seaweed-based supplements and food ingredients can be bought over the counter in Norway or on the internet. These products may contain very *high* levels of iodine, and often far more than what is declared on the packaging (31). It has been documented that the intake of such products can result in iodine excess (32). In our study, only two participants had an estimated UIE > 1,200 µg/24 h, suggesting that daily use of such very high-iodine supplements or food ingredients was not common in 2016–2017, at least not in Trøndelag county. In The National Public Health Survey 2020, 2% of adults reported having used seaweed-supplements in the last 12 months, and around 40% reported having used supplements containing iodine (19). Most people did not use these supplements daily, only 0.5% reported use of seaweed-supplements daily or most days and 20% consumed iodine-containing supplements daily or most days.

Salt iodisation is not likely to put adults in Norway at risk of iodine excess as there seems to be room for increasing the iodine intake even when the consumption of the dietary sources of iodine is high. There are, however, supplements and food ingredients available on the market with toxic levels of iodine, and caution is warranted regarding the consumption of these products.

### Strengths and limitations

Strengths of this study include the population-based design, and information was collected in the health examination survey about the most important sources of iodine and other relevant background factors.

Limitations include that there was only a single spot-urine sample available per participant for the measurement of iodine and creatinine. Due to large day-to-day variation in UIC, this measure is of limited value for characterising iodine status at the individual level. However, the median UIC in a group is recommended for defining iodine status at group level (33, 34). The WHO has also suggested that a proportion of ≥ 20% of spot-UICs lower than 50 µg/L indicates iodine deficiency (13, 35). Ideally, one should collect at least two-three spot-urine samples from a subgroup of participants. This would allow for estimation of the full distribution of average habitual UIC and the proportions that fall below the estimated average requirement and above the upper level for safe intake (16, 36). Measuring creatinine in the same urine sample allowed us to estimate 24 h UIE and iodine intake, which removes measurement error caused by hydration status at sampling and provides a somewhat better measure of iodine intake at an individual level (18).

There were limited data on food intake since the questions on diet and supplement use in the questionnaire were few and not very detailed. For example, there was no information about whether the nutrient supplements contained iodine or not. However, the questions covered the most important sources of iodine and asked specifically for quantities of different types of milk/yoghurt and for frequencies for fish intake.

## Conclusions

Iodine status was adequate in older adults, but mildly deficient in adults under 55 years according to WHO criteria in this population study from Mid-Norway. Milk intake, supplement use and use of thyroid medication are important determinants of iodine intake in Norway. The results agree with previous studies, supporting the need for measures to increase the iodine content of the Norwegian diet.
